# Clinical Outcomes of Liver Transplantation in Patients With Hepatorenal Syndrome: A Single Center Study in China

**DOI:** 10.3389/fsurg.2021.781648

**Published:** 2022-01-28

**Authors:** Fangcong Li, Tielong Wang, Liqiang Zhan, Zehua Jia, Tao Luo, Shirui Chen, Qiang Zhao, Zhiyong Guo, Xiaoshun He, Dongping Wang

**Affiliations:** Organ Transplant Center, The First Affiliated Hospital, Sun Yat-sen University, Guangzhou, China

**Keywords:** hepatorenal syndrome, HRS, acute kidney injury, AKI, liver transplantation, chronic kidney disease, CKD

## Abstract

**Background:**

Liver transplantation (LT) is an optimal treatment for hepatorenal syndrome (HRS) patients but renal function recovery is not universal after operation. The aim of this study is to explore the association between stages of hepatorenal syndrome—acute kidney injury (HRS-AKI) and incidence of post-operation chronic kidney disease (CKD).

**Methods:**

Data of HRS-AKI patients who received LT were collected from the First Affiliated Hospital of Sun Yat-sen University from 2016 to 2020. A survival and incidence curve and multivariable model were established to analyze the impacts of HRS-AKI stages and variables on 90-day survival and CKD within 12 months.

**Results:**

A total of 62 HRS-AKI patients were enrolled in this study. Overall, 35 (57%), 17 (27%), and 10 (16%) patients were diagnosed as stages 1, 2, and 3, respectively. The patients at stage 3 had the poorest outcomes with the lowest rate of 90-day survival and the highest incidence of CKD in 12 months. Stage 3 (SHR = 7.186, 95% CI, 1.661–32.043) and postoperative renal replacement therapy (RRT) (SHR = 3.228, 95% CI, 1.115–9.345) were found as useful indicators for poor prognosis.

**Conclusions:**

In our study, the classification of HRS-AKI stages can be used to predict the prognosis of HRS patients after LT. The peak serum creatinine level is a risky predictor in high HRS-AKI stage patients.

## Introduction

Hepatorenal syndrome (HRS) is a severe complication of kidney injury commonly found in patients with liver disease. Kidney dysfunction occurs in the condition of decompensated cirrhosis, acute-on-chronic liver failure, or even acute liver failure (1–3). The marked characteristics in HRS are decreased renal flow and glomerular filtration rate (GFR) with elevated serum creatinine levels in practice (1). There are two types of HRS (4). Type 1 HRS (HRS1) is characterized as rapid renal dysfunction, termed acute kidney injury (AKI). With the concept of AKI proposed in 2014, the International Club of Ascites (ICA) re-named HRS1 as hepatorenal syndrome-acute kidney injury (HRS-AKI) (5). However, the former HRS criteria based on the cut-off serum creatinine levels are still widely used in clinical practice (6–9).

For therapy strategies, several studies have reported the effects of vasoconstrictors on HRS, such as noradrenaline, midodrine, octreotide, and, in particular, terlipressin with albumin (6, 8, 10, 11). Still, liver transplantation (LT) plays a crucial role in the treatment of HRS since it can fundamentally resolve cirrhosis, portal hypertension, and liver dysfunction. Patients who received LT demonstrated an obviously superior survival rate to those who did not receive LT (12). However, renal function might not recover even after LT, implying that the severity of HRS-AKI might be associated with postoperative prognosis. For these patients, a combined liver-kidney transplantation (CLKT) may be a better choice than a single liver transplantation. The relationship between severity of HRS-AKI and postoperative prognosis is controversial because the old criteria are still being used in practice. By applying the new HRS-AKI criteria, our study aims to explore the relationship between severity of HRS-AKI and 90-day survival rate after LT and incidence of chronic kidney disease (CKD).

## Patients and Methods

### Study Population

This was a retrospective study in which patients underwent LT from 1 January 2016 to 31 December 2020 at The First Affiliated Hospital of Sun Yat-sen University. Patients that met the AKI of ICA criteria, age > 18, and had undergone orthotopic LT were first collected (1). Then, patients who had accepted CKLT, living donor transplantation, second time LT, and failed to fulfill HRS criteria were excluded.

### Definitions

The diagnostic criteria of HRS were as follows: (1) diagnosis of AKI according to the ICA criteria; (2) cirrhosis with ascites; (3) no response after 2 consecutive days of diuretic withdrawal and plasma volume expansion with albumin; (4) absence of shock; (5) no current or recent use of nephrotoxic drugs; and (6) no signs of structural injury, which is indicated by proteinuria, micro-hematuria, and/or abnormal renal ultrasonography (Table 1).

**Table 1:** Definitions of AKI by ICA and diagnostic criteria of HRS-AKI.

	**Definition**
Baseline SCr	Baseline serum creatinine (SCr) was defined as value obtained in the previous 3 months. In patients with more than one value, the one closest to the admission time should be used. In patients without, the serum creatinine on admission should be used as baseline.
AKI definition	Increase in SCr ≥0.3 mg/dl (26.5 μmol/L) within 48 h; or, A percentage increase SCr ≥50% from baseline which is known, or presumed, to have occurred within the prior 7 days
AKI stage	• Stage 1: increase in SCr ≥0.3 mg/dl (26.5 μmol/L) or an increase in SCr ≥ 1.5-fold to 2-fold from baseline • Stage 2: increase in SCr >2-fold to 3-fold from baseline • Stage 3: increase in SCr >3-fold from baseline or SCr ≥4.0mg/dl (353.6 μmol/L) with an acute increase ≥0.3 mg/dl (26.5 μmol/L) or initiation of renal replacement therapy
Diagnostic criteria of HRS-AKI	• Diagnosis of AKI according to ICA AKI criteria • Cirrhosis with ascites • No response after two consecutive days of diuretic withdrawal and plasma volume expansion with albumin • Absence of shock • No current or recent use of nephrotoxic drugs (NSAIDs, aminoglycosides, iodinated contrast media, etc.) • No macroscopic signs of structural kidney injury, as followings: • Absence of proteinuria (>500 mg/day) • Absence of microhaematuria (>50 RBCs per high power field) • Normal findings on renal ultrasonograpgy

*AKI, acute kidney injury; ICA, International Club of Ascites; HRS, hepatorenal syndrome; SCr, serum creatinine; NSAIDs, non-steroidal anti-inflammatory drugs; RBCs, red blood cells*.

The definition of HRS-AKI complied with the ICA criteria, in which AKI was categorized into three stages: stages 1, 2, and 3. Baseline serum creatinine (SCr) was defined as the value obtained in the previous 3 months. In patients with more than one value, the one nearest to the admission time was used.

The definition of CKD was based on The Kidney Disease: Improving Global Outcomes (KDIGO) clinical practice guidelines, which was an estimated glomerular filtration rate (eGFR) <60 mL/min/1.73 m^2^ in the last 3 months (13). The calculation of estimated GFR was processed with The 4-variable Modification of Diet in Renal Disease (MDRD-4) equation (14).

### Data Collection

**Figure 1:**
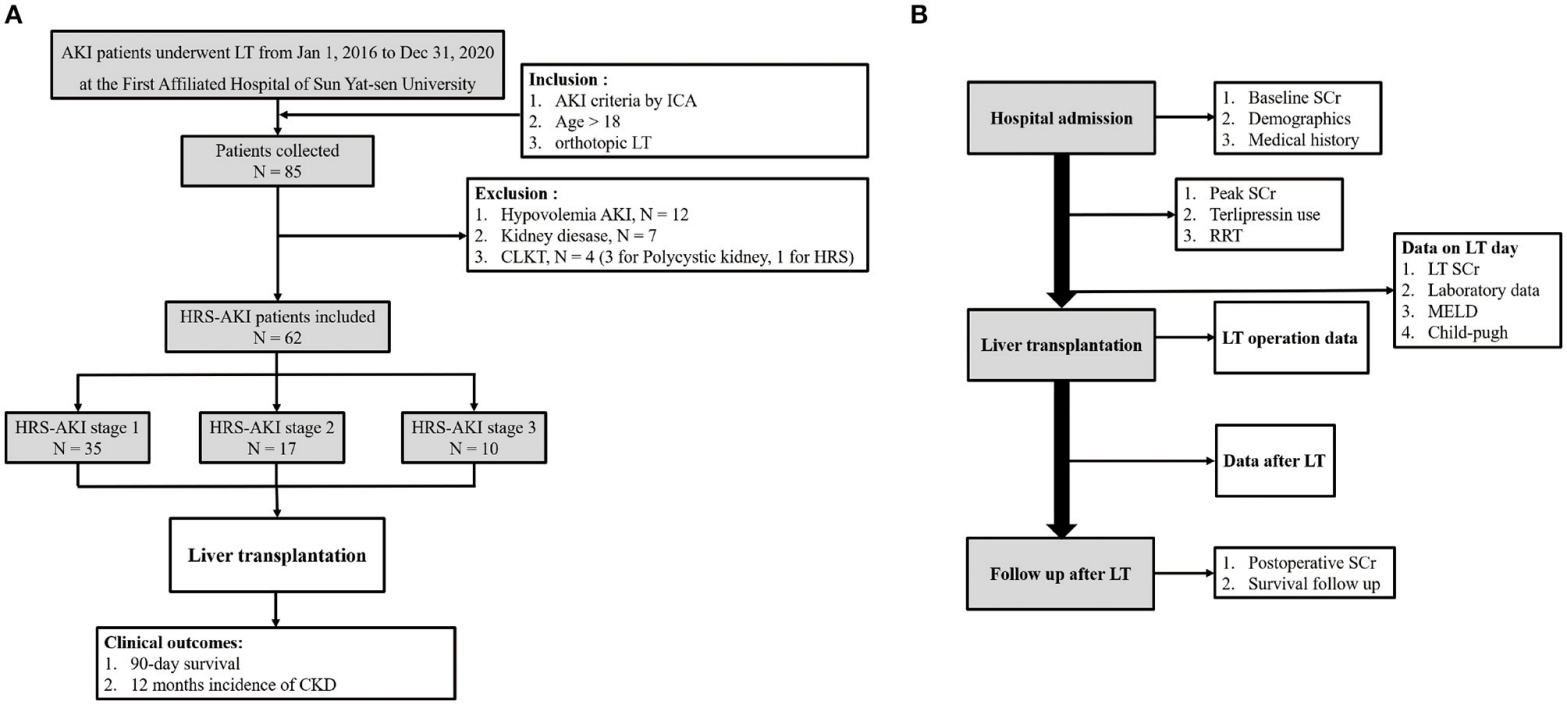
**(A)** Flow chart of study design. **(B)** Time point of data collection.

Patient demographics and clinical and laboratory data of pre-transplantation and post-transplantation were collected. Pre-transplantation variables included age, sex, etiology of cirrhosis, condition of hypertension and diabetes, hepatocellular carcinoma (HCC), terlipressin use, renal replacement therapy, baseline SCr, and peak SCr. Laboratory data, Model for End-stage Liver Disease (MELD) scores and Child-Pugh scores on the LT day were collected. Donor and LT operation information were included. Post-transplantation data were postoperative RRT, length of intensive care and hospitalization, and immunosuppressive drug use. SCr after LT and patient survival information were collected from the follow-up database of the hospital (Figure 1B).

### Statistical Analysis

On account of distribution, continuous variables were described as mean with standard deviation (m, SD) or median with interquartile range (M, IQR). For categorical variables, chi-square test was used. The one-way ANOVA test was used to compare the differences among the three groups. Survival curves were performed with the Kaplan-Meier model and a log-rank test was conducted. A Cox proportional hazards regression model was used to perform multivariate model analysis. *P* <0.05 were considered significantly different. The statistical analysis was performed using IBM SPSS Statistics 25.0 and GraphPad Prism 8.0.2.

## Results

### Patient Characteristics

From 1 January 2016 to 31 December 2020, the data of 85 AKI patients who underwent LT at the First Affiliated Hospital of Sun Yat-sen University were collected. Among these patients, 12 had hypovolemia AKI, seven had kidney disease, and four patients accepted a combined liver-kidney transplantation. Finally, 62 patients were included in the study. A total of 35 patients were at HRS-AKI stage 1 (57%), 17 (27%) were at HRS-AKI stage 2, and 10 (16%) were at HRS-AKI stage 3 (Figure 1A).

Overall, the mean age was 50 ± 12 years. There were more male (54, 87%) patients than female patients. Viral infection was the major cause of cirrhosis (49, 79%). A total of 11 (18%) patients had HCC, 15 (24%) had hypertension, and 13 (21%) had diabetes. There were no statistical differences in hypertension (*P* = 0.16), diabetes (*P* = 0.65), and HCC (*P* = 0.25) among groups. No difference was observed on their baseline SCr (77 vs. 74 vs. 83, *P* = 0.30). A significant difference was observed in the peak SCr levels before operation (118 vs. 163 vs. 275, *P* < 0.01). Terlipressin (7 vs. 11 vs. 9, *P* < 0.01) and renal replacement therapy (RRT) (4 vs. 3 vs. 9, *P* < 0.01) were investigated as meaningful variables in perioperative HRS treatment.

**Figure 2:**
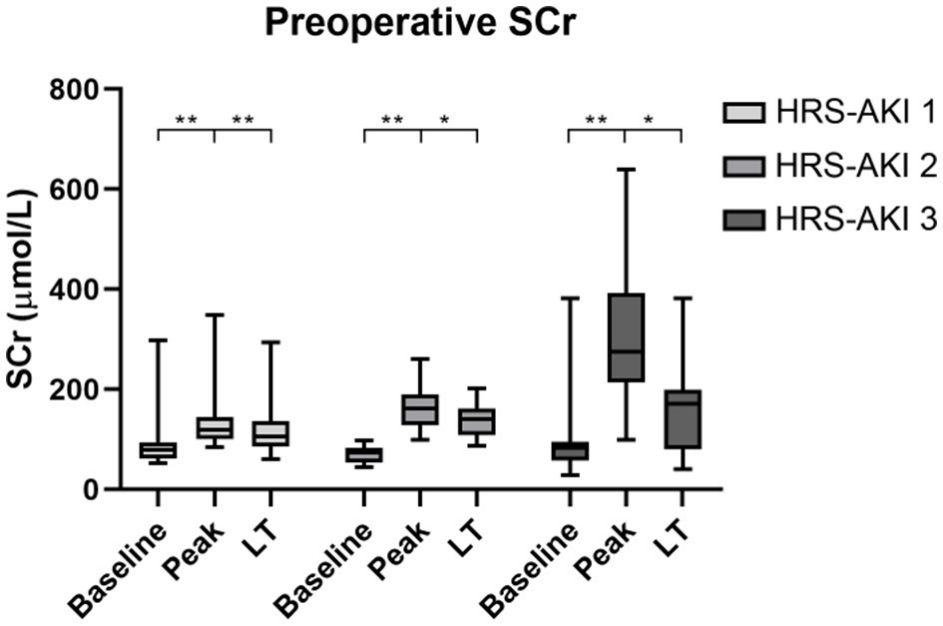
Serum creatinine levels of each HRS-AKI stage group at baseline, peak, and LT day. ^*^P < 0.05, ^**^P < 0.01.

The data on LT day (the latest data before LT) showed that the level of SCr was significantly lower compared to the peak SCr value (Figure 2). In addition, patients at HRS-AKI stage 3 had the highest WBC counts (5.5 vs. 7.5 vs. 10.9, *P* = 0.03), level of ammonia (60.1 vs. 63.3 vs. 101.2, *P* = 0.02) and Child-Pugh scores (10 vs. 11 vs. 12, *P* < 0.01). The level of SCr on LT day was also the highest, but with no meaningful difference (105 vs. 142 vs. 172, *P* = 0.08) (Table 2).

**Table 2:** Characteristics of patients in HRS–AKI groups.

	**HRS–AKI** **stage 1**	**HRS–AKI** **stage 2**	**HRS–AKI** **stage 3**	***P* value**
Patients, n	35	17	10	
Age (years), m ± SD	51 ± 11	51 ± 12	41 ± 14	0.37
Sex (male/female), n/n	33/2	16/1	5/5	<0.01
Etiology of cirrhosis, n				0.02
Viral	28	15	6	
Alcohol with/without viral	6	2	1	
Other	1	0	3	
Hypertension, n	6	7	2	0.16
Diabetes, n	8	4	1	0.65
HCC, n	8	3	0	0.25
Terlipressin use, n	7	11	9	<0.01
RRT, n	4	3	9	<0.01
Baseline SCr (μmol/L), M (IQR)	77 (62–92)	74 (55–86)	83 (58–95)	0.39
Peak SCr (μmol/L), M (IQR)	118 (100–140)	163 (129–192)	275 (214–392)	<0.01
**Data on LT day**				
Sodium (mmol/L), m ± SD	137.7 ± 5.9	136.3 ± 6.4	140.1 ± 4.4	0.27
SCr (μmol/L), M (IQR)	105 (86–127)	142 (108–165)	172 (80–200)	0.08
Urea (mmol/L), M (IQR)	10.0 (5.3–13.0)	10.1 (6.0–17.1)	9.2 (5.5–12.6)	0.80
AST (IU/L), M (IQR)	58 (33–101)	71 (31–69)	111 (61–198)	0.13
ALT (IU/L), M (IQR)	33 (21–60)	31 (15–69)	46 (25–140)	0.49
Bilirubin (μmol/L), M (IQR)	294 (54–521)	334 (124–525)	351 (266–452)	0.73
Albumin (g/L), m ± SD	38.6 ± 6.0	37.0 ± 5.2	42.1 ± 7.0	0.11
Hemoglobin (g/L), m ± SD	90.2 ± 21.2	83.6 ± 16.1	82.7 ± 14.8	0.40
WBC (×10^6^/L), M (IQR)	5.5 (3.1–8.0)	7.5 (4.4–10.7)	10.9 (7.2–19.9)	0.03
PLT (×10^6^/L), m ± SD	59 ± 29	64 ± 33	56 ± 41	0.78
INR, M (IQR)	1.94 (1.47–2.47)	2.16 (1.55–2.78)	1.83 (1.42–2.26)	0.90
Ammonia (μmol/L), m ± SD	60.1 ± 36.8	63.3 ± 40.8	101.2 ± 51.0	0.02
MELD, m ± SD	25 ± 9	29 ± 8	29 ± 9	0.19
Child–Pugh, M (IQR)	10 (9–12)	11 (9–12)	12 (12–13)	<0.01

*HRS–AKI, hepatorenal syndrome – acute kidney injury; SD, standard deviation; HBV, hepatic B virus; HCC, hepatocellular carcinoma; M, median; LT, liver transplantation; SCr, serum creatinine; IQR, interquartile range; AST, aspartate transaminase; ALT, alanine transaminase; WBC, white blood cell; PLT, platelet; INR, international normalized ratio; MELD, Model For End–Stage Liver Disease*.

There were no significant differences in the donor characteristics age, sex, BMI, donor risk index, cardiac death or brain death donation, warm ischemia time, and cold ischemia time (Table 3).

**Table 3:** Characteristics of donors.

	**HRS-AKI** **stage 1**	**HRS-AKI** **stage 2**	**HRS-AKI** **stage 3**	***P* value**
Donors, n	35	17	10	
Age (years), m ± SD	36 ± 18	36 ± 16	35 ± 18	0.98
Sex (male/female), n/n	24/11	16/1	6/4	0.08
BMI (kg/m^2^), M (IQR)	22 (20–25)	22 (19–23)	23 (20–24)	0.46
Donor risk index, M (IQR)	1.49 (1.31–1.72)	1.52 (1.30–1.81)	1.48 (1.21–1.75)	0.79
Donation after brain death, n	29	13	8	0.86
Donation after cardiac death, n	6	4	2	0.86
WIT in DCD (minutes), M (IQR)	7 (5-12)	8 (6-18)	8 (NA)	0.79
CIT (hours), M (IQR)	6 (5-8)	6 (5-9)	7 (5-8)	0.81

*HRS-AKI, hepatorenal syndrome – acute kidney injury; BMI, body mass index; SD, standard deviation; BMI, body mass index; M, median; IQR, interquartile range; WIT, warm ischemia time; DCD, donation after cardiac death; CIT, cold ischemia time*.

*Donor risk index was calculated by: https://gastro.cchmc.org/calculators/donor-risk-index/*.

No statistics differences were shown in LT operation information. All 10 patients at HRS-AKI stage 3 had undergone the piggyback technique. The comparison of significant post reperfusion syndrome occurrence among the three groups showed no meaningful discrepancy (9 vs. 7 vs. 4, *P* = 0.65).

For the outcomes of early postoperative treatment, nine patients at HRS-AKI stage 3 needed RRT and this portion was highest compared to HRS-AKI stages 1 or 2 (10 (28%) vs. 8 (47%) vs. 9 (90%), *P* < 0.01). The patients at stage 3 also had the longest duration of ICU stays (2 vs. 4 vs. 8, *P* = 0.02) since RRT needs intensive life monitoring and fluid management by ICU medical teams. The median length of postoperative hospitalization was 25 days, in which no meaningful disparity was shown among each group. There was no difference in using immunosuppressive drugs, including tacrolimus, mycophenolate mofetil, and sirolimus (Table 4).

**Table 4:** Data on LT and post LT of patients in HRS–AKI groups.

	**HRS–AKI stage 1**	**HRS–AKI stage 2**	**HRS–AKI stage 3**	***P* value**
Patients, n	35	17	10	
**LT operation data**				
Operation time (hours), M (IQR)	7 (6–8)	6 (5–8)	7 (5–8)	0.47
Blood loss (mL), M (IQR)	2500 (1500–3500)	2500 (600–4500)	1700 (1050–3625)	0.59
Anhepatic phase (minutes), m ± SD	52 ± 12	51 ± 14	51 ± 15	0.93
Piggyback technique, n	23	13	10	0.09
Significant PRS	9	7	4	0.65
**Data after LT**				
Postoperative RRT, n	10	8	9	<0.01
Length of ICU stayed (days), M (IQR)	2 (2–5)	4 (2–14)	8 (6–18)	0.02
Length of postoperative hospitalization (days), M (IQR)	22 (16–33)	29 (20–40)	28 (17–68)	0.05
Tacrolimus use, n	30	11	6	0.28
MMF use, n	20	7	6	0.62
Sirolimus use, n	6	2	3	0.42

*HRS-AKI, hepatorenal syndrome–acute kidney injury; SD, standard deviation; M, median; LT, liver transplantation; IQR, interquartile range; PRS, post–reperfusion syndrome; RRT, renal replacement therapy; ICU, intensive care unit; MMF, mycophenolate mofetil*.

### Clinical Outcomes

**Figure 3:**
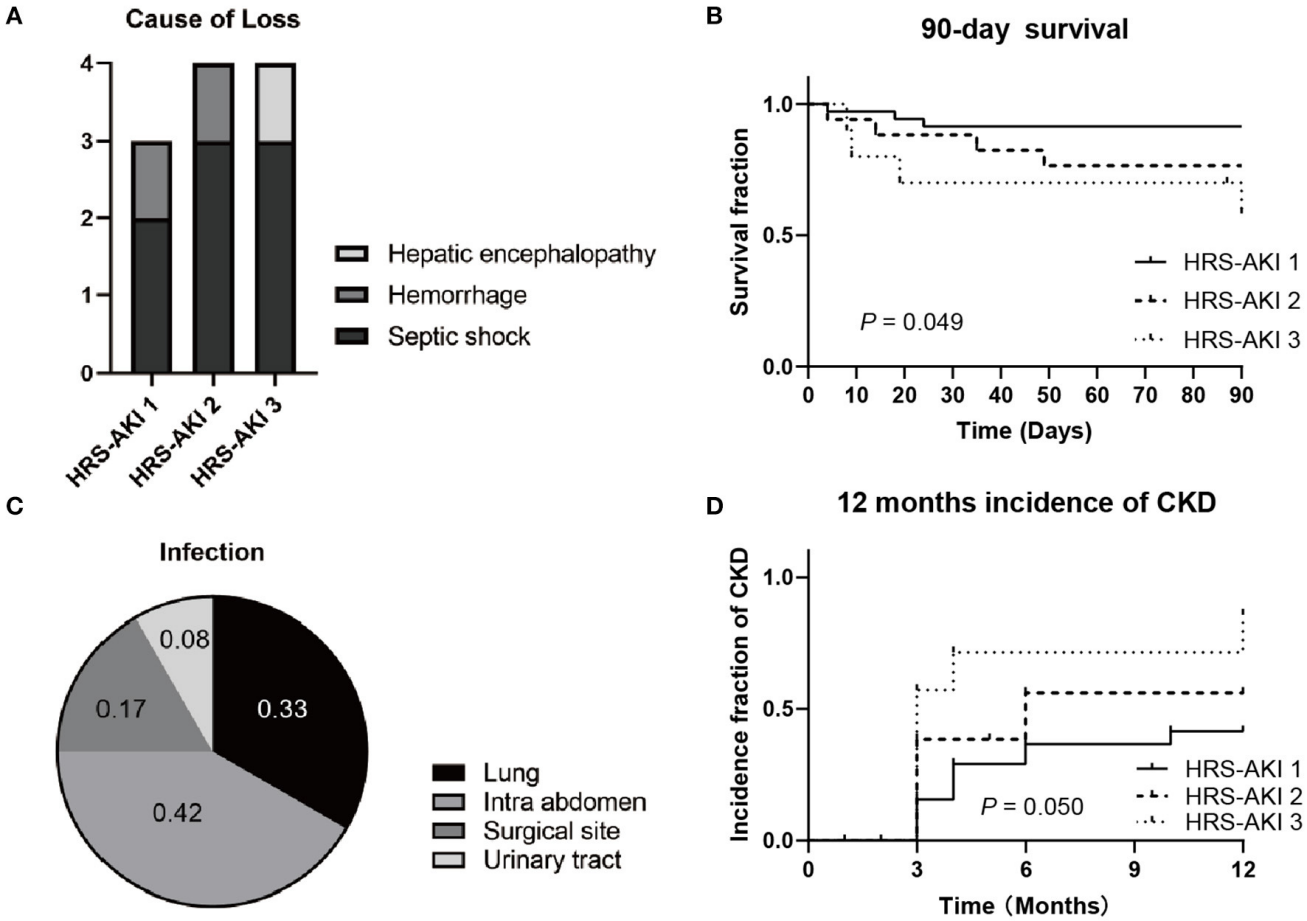
**(A)** Cause of HRS-AKI patient death at 90 days after LT. **(B)** 90-day survival curve of each stage group after LT. **(C)** Infection types of patients who died from septic shock. **(D)** CKD incidence curve of each stage group at 12 months after LT.

Data of survival demonstrated that 11 of 62 (18%) patients died 90 days after LT (Figure 3). For each group, there were 3 of 35 (9%) in HRS-AKI stage 1, 4 of 17 (24%) in HRS-AKI stage 2, and 4 of 10 (40%) in HRS-AKI stage 3 (Figure 3B). Septic shock was found to be the most common cause of death (8 of 11, 73%). Two patients died because of postoperative hemorrhage. One patient had serious hepatic encephalopathy and he did not recover even after LT (Figure 3A). The lung and intra abdomen were the major infection areas (Figure 3C). The incidence of CKD was confirmed based on a longer term of SCr level follow-up over 12 months. The accumulative incidence of CKD within 12 months among the three groups was 12 of 35 (34%, HRS-AKI stage 1), 7 of 17 (41%, HRS-AKI stage 2), and 6 of 10 (60%, HRS-AKI stage 3) (Figure 3D).

### Comparison of 90-Day Survival and Non-survival in HRS-AKI Stages 2 and 3 After LT

**Figure 4:**
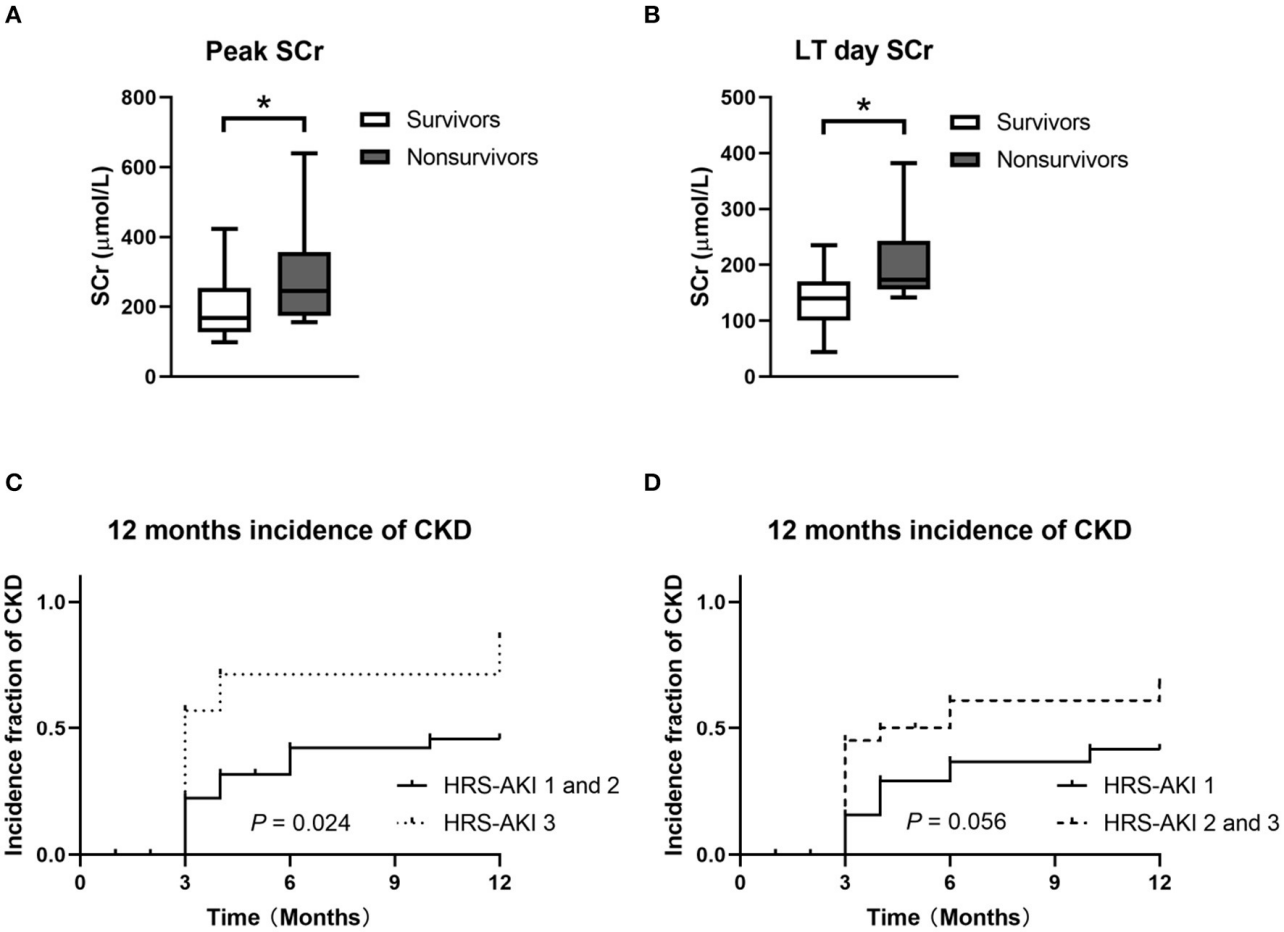
**(A,B)** Peak and LT day SCr of 90-day survivors and non-survivors in HRS-AKI stages 2 and 3. *P < 0.05. **(C)** CKD incidence curve of the HRS-AKI stage 3 group and HRS-AKI stages 1 and 2 group. **(D)** CKD incidence curve of HRS-AKI stage 1 group and HRS-AKI stages 2 and 3 group.

To analyze patient survival factors in higher HRS-AKI stages, 27 patients in HRS-AKI stages 2 and 3 were included to analyze 90-day survival. A total of 8 of 27 (30%) patients died within 90 days of LT. No significant differences were found in preoperative variables including age, sex, MELD and Child-Pugh scores, acceptance of terlipressin, and RRT. The survivors had the lower level of peak and operative-day SCr (Figures 4A,B). All the patients who died experienced RRT after LT and they also had longer stays in the ICU (4 vs. 15, *P* < 0.01) (Table 5).

**Table 5:** Comparison of 90-day survivors and nonsurvivors in HRS-AKI 2 and 3.

	**Survivors**	**Nonsurvivors**	***P* value**
Patients, n	19	8	
Age (years), m ± SD	46 ± 14	52 ± 12	0.30
Sex (male/female), n/n	15/4	6/2	0.82
Terlipressin use, n	15	5	0.63
RRT, n	8	4	0.70
MELD, m±SD	29 ± 8	31 ± 8	0.57
Child-Pugh, M (IQR)	12 (10-12)	12 (10-13)	0.83
Postoperative RRT, n	9	8	0.01
Length of ICU stayed (days), M (IQR)	4 (2–6)	15 (9–20)	<0.01

*HRS-AKI, hepatorenal syndrome – acute kidney injury; SD, standard deviation; MELD, Model For End-Stage Liver Disease; IQR, interquartile range; RRT, renal replacement therapy; ICU, intensive care unit*.

### Multivariate Model Analysis

The incidence of CKD showed no statistical difference when directly compared among the three groups (*P* = 0.05). HRS-AKI stages 1 (34%) and 2 (41%) had a relatively similar incidence and then they were combined as one group (HRS-AKI stages 1 and 2) to conduct a comparison with group HRS-AKI stage 3, which showed a meaningful result (*P* = 0.024) (Figures 4C,D).

HRS-AKI stage 3 (SHR = 7.186, 95% CI, 1.661–32.043, *P* = 0.010), age (SHR = 1.044, 95% CI, 1.007–1.083, *P* = 0.020), and postoperative RRT (SHR = 3.228, 95% CI, 1.115–9.345, *P* = 0.031) were found to be significant predictors for the occurrence of CKD within 12 months. Preoperative RRT had no significant impact on CKD (SHR = 0.237, 95% CI, 0.045–1.239, *P* = 0.088).

## Discussion

Since the emergence of the concept of HRS-AKI provided a new definition of HRS, several studies have suggested updates to challenge the traditional points. HRS is not just a purely renal functional disease but a comprehensive pathophysiological pattern. The classical visceral vasodilation theory is not able to fully explained it either (3). From clinical biopsies and an animal model of HRS, renal parenchymal damage was found to exist in practice (15–18). A retrospective study reported that only 75.8% of HRS patients recovered from renal dysfunction after LT (7). Risk factors were reported, including a higher SCr level, a longer duration of HRS, and a longer duration of dialysis.

We analyzed risk factors of HRS-AKI to help surgeons improve LT prognosis and determine whether a CLKT is necessary for HRS patients. In general, indications of CLKT include two main parts: decompensated cirrhosis and terminal renal disease. However, the fact that the renal function of HRS patients can recover after LT puts forth the question: is a donor kidney wasted for HRS patients accepting CLKT? Besides, no studies reported the advantages of a better graft and survival situation in patients who received CLKT or LT alone (19, 20). For HRS patients with unrecoverable renal function, prolonged dialysis brings a heavy burden and deteriorates quality of life. Some other predictors should be found to resolve the challenges in accurately assessing the irreversibility or progression of renal functional failure in HRS.

In our study, patients at HRS-AKI stage 3 had the worst 90-day survival rate and the highest cumulative incidence of CKD. But there was no specific difference between stage 1 and stage 2 in our study, this needs a further larger sized clinical study to investigate. Vasoconstrictors use and volume expansion, especially terlipressin plus albumin, are the effective pharmacological therapies. Our result showed that terlipressin used in HRS-AKI was prevalent, but those patients who responded to terlipressin before LT could have a lower incidence of post-transplantation CKD (21). RRT is a supportive treatment to sustain patients on the liver waiting list before they receive a time window for accepting LT. Although, no sufficient evidence proved that post-transplantation survival improved (22). HRS-AKI stage 3 had the highest rate of patients undergoing pre-LT and post-LT RRT, which also resulted in the longest duration of ICU stays. It could be considered as an indicator of poor outcomes, when the longer duration of RRT means the delayed or weak functional of the organ.

The role of inflammation and bacterial translocation is an important mechanism of HRS. The highest level of WBC counts and ammonia were found in HRS-AKI stage 3. Besides, septic shock caused eight of 11 deaths at 90 days and 42 % of infectious sources came from the intra abdomen. The gut is the major source of blood ammonia where unabsorbed protein is resolved by bacteria (23). Also, a rodent model of cirrhosis showed that kidney injury alleviated under the selective gut decontamination by antibiotic pre-use (16). Bacterial infection, especially spontaneous peritonitis, is the major precipitant triggering HRS over excess diuretics, gastrointestinal bleeding, and large-volume paracentesis according to research in 2015 (7). Above these, intestinal bacteria participate in the whole process of HRS and a further clinical study needs to study the benefit of bacterium management before LT in HRS patients.

All 10 patients in HRS-AKI stage 3 received LT *via* the piggyback technique from our data. The classic cava replacement LT technique requires the total clamping of the inferior vena cava, which interrupts venous return and brings hemodynamic changes. This may lead to development of renal injury but this suggestion is still controversial. A retrospective study showed that there was no difference of overall recovery of renal function and survival rates among these LT techniques (24). The team suggested that the amount of transfused red blood cells can be used as a predictor because it correlates to the severity of disease and the complexity of the LT surgery. However, this study did not include high-MELD candidates, so the kidney outcomes of HRS-AKI patients in pre-transplantation remain unclear. Further studies need to investigate whether different LT techniques influence postoperative kidney function of HRS patients.

Immunosuppression drugs are thought to be factors causing CKD. Tacrolimus (TAC) is a widely used calcineurin inhibitor (CNI). The use of TAC did not show a significant difference on the occurrence of CKD stage 3 compared to stages 1 and 2. A recent study on longitudinal TAC exposure found that TAC was not a predictor for CKD after LT at 12 months and that HRS-AKI increased the risk of CKD (25). Moreover, the authors also asked the question of reversal of renal function in HRS-AKI after LT, suggesting that some HRS cases do not constitute irreversible renal injury. This view is consistent with the present study, where HRS-AKI stage 3 showed the highest incidence of CKD. It suggests the implication that patients developed to stage 3 have renal solid organ damage.

There were limitations in this study based on its retrospective nature. Firstly, the admission SCr level used as baseline was substituted when the one before admission was missed. The higher baseline could make the diagnosis of HRS-AKI conservative. Secondly, the small number of patients caused unavoidable biases on the results and multivariate model analysis. Thirdly, HRS patients who accepted CLKT should be included as a group to study, but the number in our center can hardly achieve this goal.

## Conclusion

In our study, classification of HRS-AKI stages can be used to predict the prognosis of HRS patients after LT. HRS-AKI stage 3 is associated with the poorest 90-day survival rate and highest incidence of CKD. For patients at HRS-AKI stages 2 and 3, the level of preoperative serum creatinine is the key impact factor for prognosis after LT.

## Data Availability Statement

The raw data supporting the conclusions of this article will be made available by the authors, without undue reservation.

## Ethics Statement

The studies involving human participants were reviewed and approved by the First Affiliated Hospital of Sun Yat-sen University. Written informed consent for participation was not required for this study in accordance with the national legislation and the institutional requirements.

## Author Contributions

FL and TW contributed to the conception and design of the study. DW, XH, and ZG gave administrative support. DW, XH, ZG, and QZ gave provision of study materials and patients. LZ, TL, and SC collected and assembled data. FL and ZJ performed data analysis and interpretation. All authors took in manuscript writing and gave final approval of the manuscript.

## Conflict of Interest

The authors declare that the research was conducted in the absence of any commercial or financial relationships that could be construed as a potential conflict of interest.

## Publisher's Note

All claims expressed in this article are solely those of the authors and do not necessarily represent those of their affiliated organizations, or those of the publisher, the editors and the reviewers. Any product that may be evaluated in this article, or claim that may be made by its manufacturer, is not guaranteed or endorsed by the publisher.

## Abbreviations

HRS: hepatorenal syndrome
GFR: glomerular filtration rate
AKI: acute kidney injury
ICA: International Club of Ascites
HRS-AKI: hepatorenal syndrome
LT: liver transplantation
CLKT: combined liver-kidney transplantation
SCr: serum creatinine
CKD: chronic kidney disease
KDIGO: The Kidney Disease: Improving Global Outcomes
MDRD: Modification of Diet in Renal Disease
HBV: hepatitis B virus
ACLF: acute-on-chronic liver failure
HCC: hepatocellular carcinoma
RRT: renal replacement therapy
ICU: intensive care unit
TAC: tacrolimus.
